# Impact of switching from the CKD-EPI_2009_ to the EKFC equation on the epidemiology of chronic kidney disease and nephrology workload

**DOI:** 10.1093/ckj/sfaf278

**Published:** 2025-09-08

**Authors:** Priscila Villalvazo, Luis Miguel Molinero-Casares, Alberto Ortiz

**Affiliations:** Department of Nephrology and Hypertension, IIS-Fundacion Jimenez Diaz UAM, Madrid, Spain; ISCIII RICORS2040; Madrid, Spain; Alce Ingeniería, Madrid, Spain; Department of Nephrology and Hypertension, IIS-Fundacion Jimenez Diaz UAM, Madrid, Spain; ISCIII RICORS2040; Madrid, Spain; Departamento de Medicina, Facultad de Medicina, Universidad Autónoma de Madrid, Madrid, Spain

**Keywords:** albuminuria, chronic kidney disease, CKD-EPI, EKFC, epidemiology

## Abstract

**Background:**

Chronic kidney disease (CKD) can be diagnosed by estimating the glomerular filtration rate (eGFR) using serum creatinine-based equations, mainly CKD-EPI_2009_. The European Federation of Clinical Chemistry and Laboratory Medicine (EFLM) recently supported adopting the European Kidney Function Consortium (EKFC) equation.

**Methods:**

We compared eGFR values obtained using CKD-EPI_2009_ and EKFC in analytical records from a single laboratory corresponding to 216 637 individual adults receiving primary and specialized healthcare in 2023 in a catchment area in Madrid (Spain).

**Results:**

Switching from CKD-EPI_2009_ to EKFC resulted in a 39.6% higher prevalence of low eGFR consistent with CKD G3-G5 (13.4% vs 9.6% in the full population and 36.3% vs 25.6% among people aged ≥65 years). Among 33 789 patients with albuminuria assessments, the prevalence of eGFR and albuminuria values consistent with CKD G1-G5 was 36.3% for EKFC and 32.5% for CKD-EPI_2009_. Among newly identified patients with potential CKD G3–G5, 25% had A2–A3 albuminuria. Differences in CKD prevalence between both equations were observed for men and women. According to National guidelines, in the first year after the switch, 0.33 to 0.58 full-time nephrologist equivalents would be needed to care for newly diagnosed patients with CKD in this catchment area, potentially resulting in between five and nine nephrologists needed for the Madrid region.

**Conclusion:**

In this retrospective analysis, a switch from the creatinine-based CKD-EPI_2009_ to the EKFC eGFR equation would increase the prevalence of CKD, especially among the elderly. EKFC may identify patients with A2–A3 albuminuria as having CKD that may have been missed by CKD-EPI_2009_ in healthcare systems with low uptake of albuminuria assessments.

KEY LEARNING POINTS **What was known**:

- The KDIGO 2024 Clinical Practice Guideline for CKD recommends using the same validated GFR-estimating equation to derive eGFR from serum filtration markers within geographical regions as large as possible.
- CKD-EP_I2009_ has so far been used in Europe while the USA recently switched to CKD-EPI_2021_.
- The European Federation of Clinical Chemistry and Laboratory Medicine (EFLM) supports switching to the European Kidney Function Consortium (EKFC) equation in Europe.
- However, the consequences of such switch on the epidemiology of CKD and on nephrology workload are poorly understood.

**This study adds**:

- A switch from the creatinine-based CKD-EPI_2009_ to the EKFC eGFR equation would increase the prevalence of CKD, especially among the elderly.
- EKFC may identify patients with A2–A3 albuminuria as having CKD and requiring therapy that may have been missed in healthcare systems that use CKD-EPI_2009_.coexisting with a low uptake of albuminuria assessment.
- In the first year after the switch, five to nine full-time nephrologists are estimated to be needed to care for newly diagnosed patients with CKD according to National guidelines in the region of 7 million people.

**Potential impact**:

- A switch from the creatinine-based CKD-EPI_2009_ to the EKFC eGFR equation would modify the epidemiology of CKD, especially among the elderly.
- This would allow the earlier identification of patients with kidney injury.
- However, the switch may cause an abrupt increase in the demand for nephrology care.

## INTRODUCTION

Chronic kidney disease (CKD) is among the fastest growing global causes of death [1]. By 2050, it is forecasted to become the third most common cause of death in countries with long life expectancy, such as Western Europe and Japan [2]. The growth of CKD burden is mainly driven by population aging, as the forecasted 140% increase in all-age deaths from CKD by 2050 becomes 33% when adjusted for age [2]. CKD is defined as abnormalities of kidney structure or function, present for >3 months, with implications for health [3, 4]. Two key quantitative criteria allow diagnosing and risk stratifying CKD and initiating cardiorenal protective therapy: glomerular filtration rate, usually estimated (eGFR) from serum creatinine, and albuminuria, expressed as the urinary albumin: creatinine ratio (UACR). The CKD-EPI_2009_ equation has been widely adopted globally to estimate eGFR from serum creatinine, adapted to local population characteristics in some regions [5, 6]. In 2021, US physicians, concerned by the presence of race in CKD-EPI_2009_, generated a race-free equation, CKD-EPI_2021_ [7]. In Europe, race data are frequently not collected in administrative databases and CKD-EPI_2009_ is reported in a race-free manner by clinical biochemistry laboratories [8]. Furthermore, the CKD-EPI_2021_ equation overestimates GFR in self-described non-Black individuals, being less accurate than CKD-EPI_2009_. Non-Black represents most of the European population [4, 8]. CKD-EPI_2021_ also underestimates GFR in self-described Black individuals as compared to CKD-EPI_2009_. This was deemed an advantage, favoring earlier diagnosis and treatment of CKD [8]. The Kidney Disease: Improving Global Outcomes (KDIGO) 2024 Clinical Practice Guideline for the Evaluation and Management of CKD recommends using a validated GFR-estimating equation to derive eGFR from serum filtration markers [4], using the same equation within geographical regions as large as possible. CKD-EPI_2009_, CKD-EPI_2021_, and the European Kidney Function Consortium (EKFC) equation are considered validated. EKFC was endorsed by the European Federation of Clinical Chemistry and Laboratory Medicine (EFLM) [9]. Compared to CKD-EPI2009 or CKD-EPI2021, EKFC showed smaller bias and higher P30 (percentage of estimated values within 30% of measured GFR) [10].

Considering a switch of eGFR equations in routine clinical care from CKD-EPI_2009_ to EKFC requires understanding its impact on individuals and the healthcare system. We have now analyzed the impact of switching from CKD-EPI_2009_ to EFKC on the epidemiology of CKD in a large contemporary dataset from Southern Europe, taking advantage of Madrid being the European region with the longest life expectancy [11] to generate data that may be interesting for healthcare systems and policymakers to plan for future care as populations worldwide are growing older [12, 13]. We also provide a rough estimate of the impact of the equation switch on nephrology workloads and workforce needs.

## MATERIALS AND METHODS

### Study design and data processing

This was an observational, retrospective, cross-sectional study performed at a single clinical laboratory providing all assessments for primary care and specialist outpatients in a single healthcare catchment area, including those at the Jiménez Díaz Foundation (FJD) University Hospital, from 1 January to 31 December 2023 were included. In Spain, the public healthcare system assigns catchment areas to hospitals based on home addresses. According to official regional records, the FJD catchment area covers 451 269 people [14] and all laboratory samples are processed in a single laboratory. Only the last available assessment for each individual was analyzed. CKD was considered potentially present since only a single data point was analyzed per patient. This approach allows addressing the aim of the study, which was to compare various creatinine-based eGFR equations. An eGFR ≤60 ml/min/1.73 m^2^ or UACR ≥30 mg/g regardless of the GFR value were considered to represent potential CKD.

Data was automatically extracted from analytical reports by randomly assigning a unique identification number to maintain anonymity. Demographic characteristics and comorbidities (hypertension, diabetes) were collected from electronic health records. Data processing included the exclusion of extreme values (age <18 or >105 years, analytical values considered to be clinically implausible). Only records with data on creatinine ≥0.4 mg/dl were analyzed. eGFR was recalculated from serum creatinine, sex, and age using CKD-EPI_2009_ without race correction and EKFC [5, 15–17] (Supplementary methods).

### Estimation of nephrology workload

The nephrology workload was estimated from the 2022 Spanish consensus guidance documents for nephrology referral based on the KDIGO risk heatmap [18] (details in Supplementary Methods). The primary care workload for newly diagnosed CKD patients was not estimated because all participants were healthcare users with laboratory assessments in the public health systems and, thus, were already followed by primary care, which is the port of entry for specialized care.

### Ethical aspects

The study was approved by the Ethics Committee of the Autonomous University of Madrid (EO014-22_FJD) and conducted in accordance with the Declaration of Helsinki (Edinburgh, 2000) and its subsequent updates.

### Statistical analysis

Quantitative variables were expressed as median (interquartile range, IQR) and qualitative variables as frequency and percentage. eGFR values obtained with CKD-EPI_2009_ and EKFC were compared by linear regression analysis. Pearson correlation coefficients were calculated to quantify the strength of the relationship between formulas. Concordance between eGFRs was evaluated using a Bland–Altman graph. Statistically significant differences between categorical values were evaluated by the chi squared or Fisher’s exact tests, as appropriate. Differences between numerical values were assessed using Student’s *t*-test for normally distributed variables, and the non-parametric Mann–Whitney *U*-test for non-normal distributions. A *P* ≤ .05 was considered statistically significant. The statistical package R version 4.2.1 was used. eGFR trajectory graphs were made with the Pandas statistical package in Jupyter Notebook (Anaconda 3) using Python programming language version 3.13.1.

## RESULTS

### Cohort description

A total of 216 637 records were assessed, corresponding to individual adult users of the healthcare system that had outpatient biochemistry laboratory assessments during 2023. This contemporary cohort represented 48.0% of the population of 451 269 people assigned to the catchment area [14]. Population characteristics are summarized in Table 1. Median age was 56 years (IQR 41–70 years). The age distribution is shown in Fig. S1. Most (126 793, 58.5%) were women. Men and women differed in multiple analytical variables. Despite similar age, men had lower UACR values but higher prevalence of hypertension (27.5% vs 21.8%, *P* < .0001) and diabetes (12.1% vs 7.3%, *P* < .0001). Missing values are shown in Table S1.

**Table 1:** Clinical and laboratory values of the study population.

Variable	All (*n* = 216 637)	Men (*n* = 89 844)	Women (*n* = 126 793)	*P* value (men vs women)
Age (years)	56 (41–70)	56 (42–69)	56 (41–70)	.000 162
Women, *n* (%)	126 793 (58.5)			
Hypertension, *n* (%)	52 433 (24.2)	24 708 (27.5)	27 725 (21.8)	<.0001
Diabetes, *n* (%)	20 238 (9.3)	10 877 (12.1)	9361 (7.3)	<.0001
CKD G3-G5				
According to CKD-EPI_2009_, *n* (%)	20 846 (9.6)	8721 (9.7)	12 125 (9.5)	<.0001
According to EKFC, *n* (%)	29 140 (13.4)	11 564 (12.9)	17 576 (13.7)	<.0001
CKD G1-G5^a^				
According to CKD-EPI_2009_, *n* (%)	24 358 (11.2)	10 690 (11.9)	13 668 (10.8)	<.0001
According to EKFC, *n* (%)	32 230 (14.9)	13 324 (14.8)	18 906 (14.9)	<.0001
Dialysis, *n* (%)	364 (0.2)	237 (0.3)	127 (0.1)	<.0001
Specialty care, *n* (%)	16 848 (7.8)	7 793 (8.7)	9055 (7.1)	<.0001
Glucose (mg/dl)	92 (85–102)	95 (87–106)	90 (83–99)	<.0001
Creatinine (mg/dl)	0.8 (0.7–1.0)	1.0 (0.9–1.1)	0.8 (0.7–0.8)	<.0001
eGFR CKD-EPI_2009_ (ml/min/1.73 m^2^)	88.3 (74.7–100.9)	86.9 (73.9–98.7)	89.3 (75.4–102.5)	<.0001
eGFR EKFC (ml/min/1.73 m^2^)	83.0 (69.6–96.3)	82.6 (69.9–94.7)	83.4 (69.5–97.4)	<.0001
UACR (mg/g)	8.3 (4.4–22.8)	7.8 (4.0–25.5)	8.7 (4.9–20.8)	<.0001
HbA1c (%)	5.5 (5.2–5.9)	5.5 (5.2–6.0)	5.4 (5.1–5.8)	<.0001

a Albuminuria data were only available in 33 789 participants.

### eGFR estimation using CKD-EPI_2009_ and EKFC

**Figure 1:**
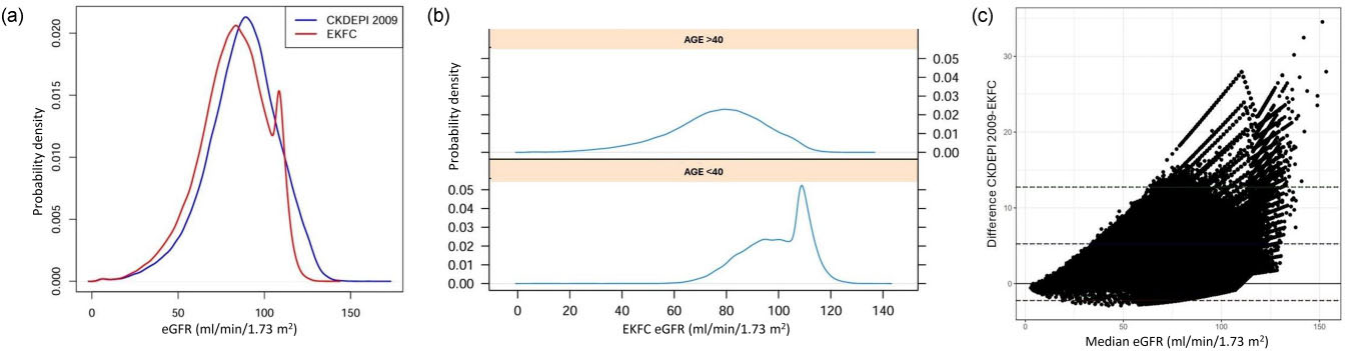
Probability density and Bland–Altman graphs comparing CKD-EPI_2009_ and EKFC eGFR values. (**a**) Probability density of eGFR values obtained using CKD-EPI_2009_ and EKFC. (**b**) EKFC eGFR according to age. The peak observed in the EKFC probability density line in panel (a) corresponds to people aged 40 years or younger. (**c**) Bland–Altman graph showing CKD-EPI_2009_ and EKFC eGFR.

EKFC eGFR values were lower than CKD-EPI_2009_ values (Fig. 1a). EKFC had a density peak at >110 ml/min/1.73 m^2^ corresponding to people aged 40 years or younger (Fig. 1b). In the Bland–Altman plot, most data points have positive differences, consistent with higher CKD-EPI_2009_ than EKFC eGFR values. Differences were larger in the higher eGFR range (Fig. 1c).

**Figure 2:**
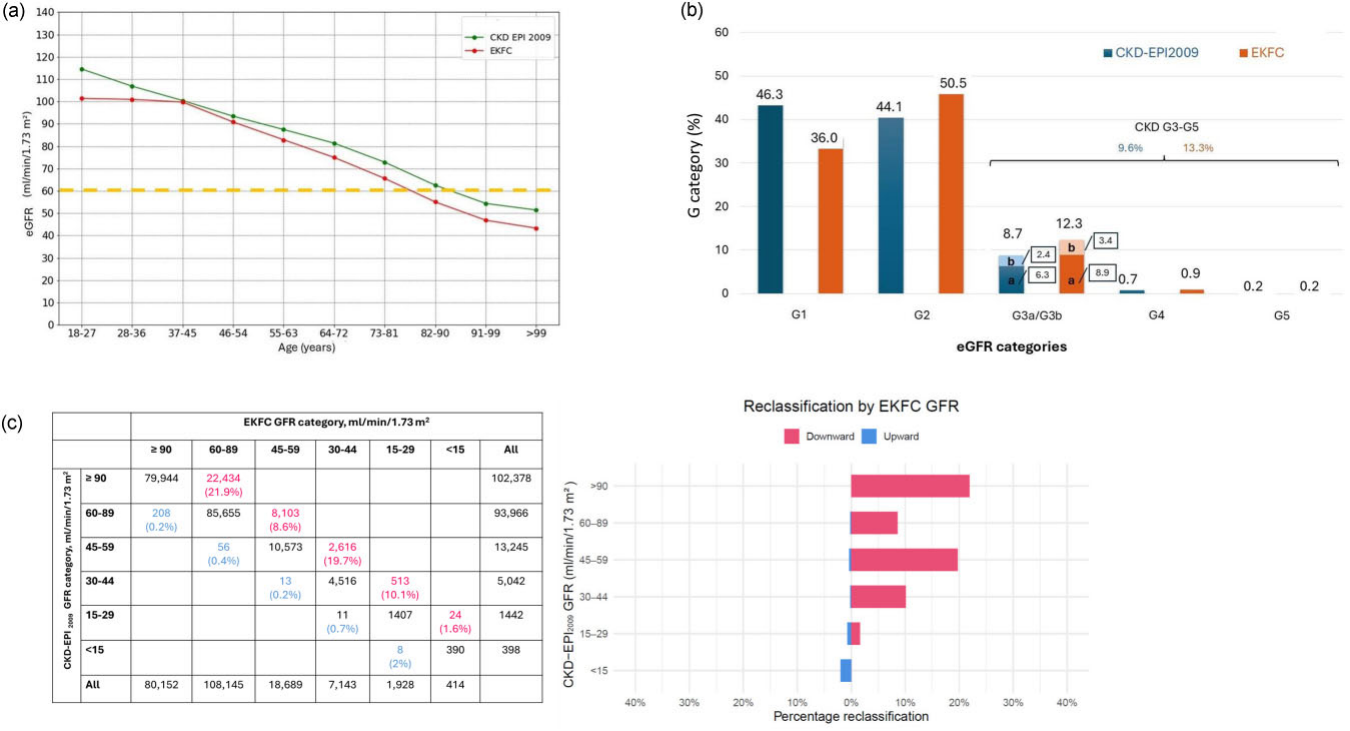
Median eGFR values and prevalence of KDIGO G categories for CKD-EPI_2009_ and EKFC equations in the full population. (**a**) Median eGFR values according to age in the full dataset when eGFR was estimated using the CKD-EPI_2009_ and EKFC equations. They were higher for CKD-EPI_2009_ than for EKFC (*P* < .0001). (**b**) Prevalence of KDIGO G risk categories and of CKD G3-G5 in the full dataset. Prevalence values are based solely on eGFR-based CKD staging. (**c**) Reclassification table.

CKD-EPI_2009_ eGFR progressively decreased from the youngest to the oldest age category (Fig. 2a). By contrast, EKFC eGFR was stable up to age 40 years and decreased thereafter. Consequently, CKD-EPI_2009_ eGFR values were higher than EKFC values in individuals under 40 years of age, approximately equal around age 40, and progressively higher thereafter up to age 64, both in men and women (Fig. S2). Median eGFR values were below the threshold to diagnose CKD (60 ml/min/1.73 m^2^) for people aged ≥91 years for CKD-EPI_2009_ and from a decade earlier (≥82 years) for EKFC.

### EKFC eGFR results in a higher prevalence of CKD G3–G5 than CKD-EPI_2009_

CKD G3–G5 (eGFR <60 ml/min/1.73 m^2^) may be diagnosed without available UACR values. The prevalence of EKFC eGFR and CKD-EPI_2009_ eGFR values consistent with CKD G3–G5 was assessed in the full study population.

The prevalence of CKD G3–G5 was higher for EKFC (13.4%) than for CKD EPI_2009_ (9.6%) (Fig. 2b). EKFC resulted in lower prevalence of the G1 risk category and a higher prevalence of other G categories. In absolute terms, patients moved from G1 (which lost 10.1 percentage points) with CKD-EPI_2009_ to EKFC categories G2 and G3 (which gained a combined 10.2 percentage points). In relative terms, the prevalence of G3 was 41% higher using EKFC compared to CKD-EPI_2009_: A similar pattern was observed in men and women, although the magnitude of change differed by sex (Figure S3). A reclassification table summarizes the results (Fig. 2c).

**Figure 3:**
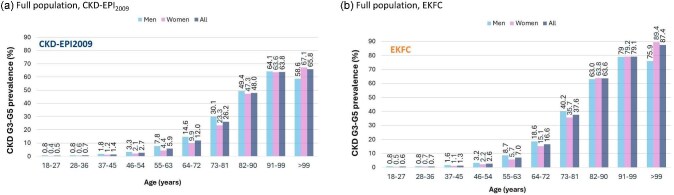
Prevalence of CKD G3-G5 for CKD-EPI_2009_. and EKFC equations in the full population, by sex and age. (**a**) CKD-EPI_2009_ equation. (**b**) EKFC equation.

The prevalence of CKD G3-G5 increased with age, especially from 55+ years, peaking at 64% in men in their nineties and 67% in centenarian women with CKD-EPI_2009_ (Fig. 3a) and at 79% and 89%, respectively, with EKFC (Fig. 3b). Among people older than 65 years, the prevalence of CKD G3–G5 was 36% for EKFC and 25% for CKD-EPI_2009_: 35% and 25%, respectively, for men and 38% and 26%, respectively, for women. Figure S4 shows KDIGO G risk categories and prevalence of CKD G3–G5 in the full study population according to sex and age.

### Laboratory characteristics of patients with CKD G3–G5

Table 2 presents summary laboratory data for patients with CKD G3–G5 according to CKD-EPI_2009_ or EKFC, Table S2 for the individual G3, G4, and G5 categories and Table S3 for G3–G5 in those older than 65 years. Overall, patients categorized as having CKD G3-G5 based on EKFC were more frequently women and had milder laboratory abnormalities potentially associated to CKD and comorbidities than those categorized as having CKD G3–G5 based on CKD-EPI_2009_.

**Table 2:** Clinical and laboratory values of participants with CKD G3-G5.

Variable	CKD G3-G5 EPI2009 (*n* = 20 846)	CKD G3-G5 EKFC (*n* = 29 140)	*P* value	CKD G3-G5 EKFC but not CKD-EPI2009 (*n* = 8344)
Age (years)	80.0 (72.0–87.0)	80.0 (72.0–87.0)	.338	79 (72–85)
Women, *n* (%)	12125 (58.2)	17 576 (60.3)	<.0001	5459 (65.4%)
Diabetes, *n* (%)	4905 (24.5)	6229 (22.4)	<.0001	1324 (16.8%)
Specialty care, *n* (%)	3751 (18.0)	4751 (16.3)	<.0001	1003 (12%)
Glucose (mg/dl)	100.0 (90.0–115.0)	99.0 (90.0–113.0)	<.0001	98.0 (89.0–110.0)
Creatinine (mg/dl)	1.2 (1.0–1.4)	1.1 (0.9–1.3)	<.0001	0.9 (0.8–1.0)
eGFR CKD-EPI_2009_ (ml/min/1.73 m^2^)	50.1 (41.0–55.8)	54.8 (45.2–60.7)	<.0001	63.0 (61.5–64.9)
eGFR EKFC (ml/min/1.73 m^2^)	45.6 (37.5–51.0)	50.1 (41.3–55.7)	<.0001	57.4 (55.8–58.8)
UACR (mg/g)	24.9 (9.6–91.9)	21.7 (8.7–76.7)	.002	11.9 (6.4–30.1)
HbA1c (%)	5.8 (5.4–6.3)	5.7 (5.4–6.3)	<.0001	5.7 (5.4–6.1)

Some have proposed that patients older than 65 years with CKD G3a should not be considered to have CKD unless there is other evidence of CKD, while people younger than 40 years should be diagnosed with CKD when eGFR is 60–75 ml/min/1.73 m^2^, the so-called age-adapted eGFR threshold for low eGFR/CKD [19]. Among patients older than 65 years, the prevalence of category G3a was 43% higher when assessed by EKFC than when assessed by CKD-EPI_2009_ and the 4939 patients newly diagnosed with CKD category G3a represented 64% of the newly diagnosed patients with CKD G3–G5 (Table S4).

### Prevalence of CKD G1–G5 among participants with albuminuria values

In 33 789 patients (15.5% of the total population) eGFR and albuminuria data were available (Table S5). and the prevalence of UACR and eGFR values consistent with CKD G1–G5 categories could be estimated. Using CKD-EPI_2009_, 7189 participants had CKD G3–G5, representing 21.2% of all patients with CKD G3–G5 in the full cohort. Using EKFC, there were 9165 participants with CKD G3–G5 (28.4% of the full CKD G3–G5 population). This suggests a bias toward higher risk of CKD G3–G5 in the population that had albuminuria data that was more evident with EKFC.

**Figure 4:**
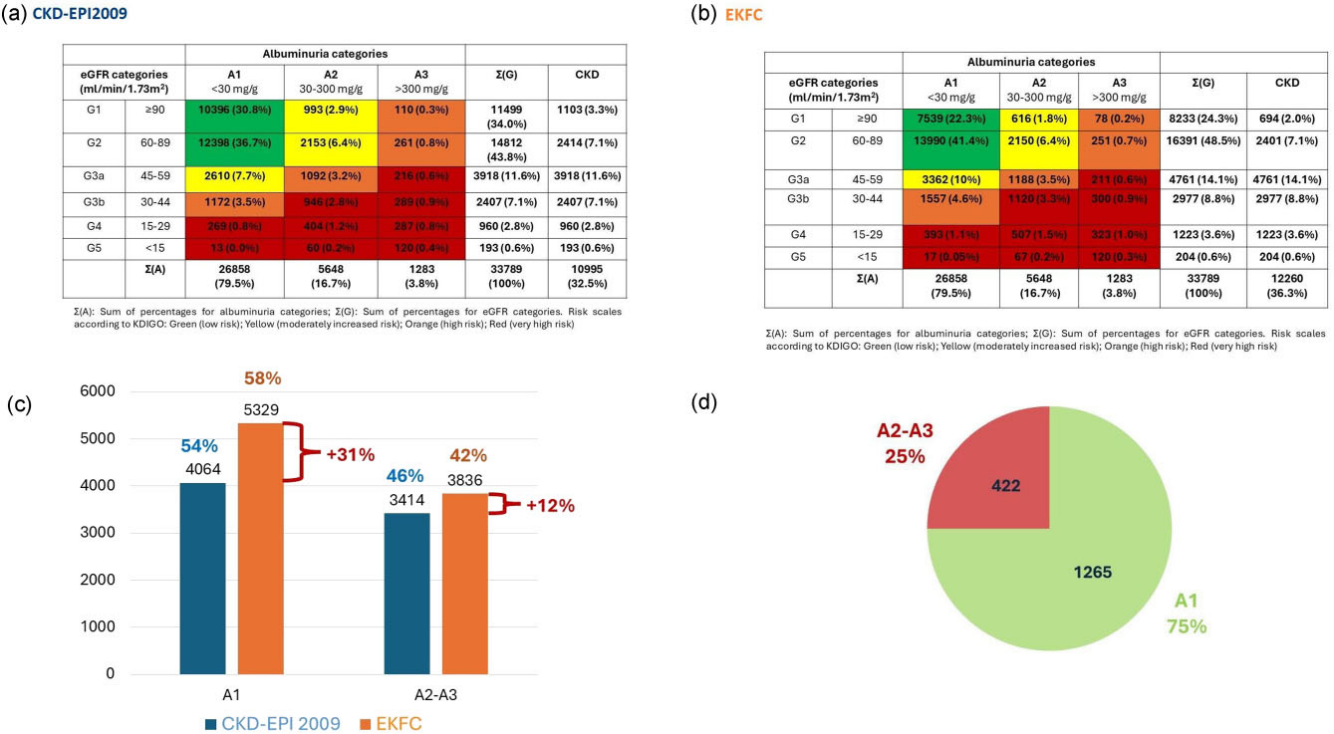
Prevalence of CKD and distribution of KDIGO risk categories for CKD-EPI_2009_. and EKFC equations in the population with UACR values. (**a**) eGFR G categories and albuminuria A categories for CKD-EPI_2009_. (**b**) eGFR G categories and albuminuria A categories for EKFC. (**c**) When switching from the CKD-EPI_2009_ to the EKFC equating, the largest increase in the CKD G3-G5 population was observed in the A1 category. The percentage increase in CKD G3-G5 patients is shown in red font and brackets. Color-coded percentages above each column represent the percentage of patients in each albuminuria category for the 100% of patients with CKD G3-G5 according to CKD-EPI_2009_ (blue) or EKFC (orange). (**d**) Distribution of KDIGO albuminuria (A) categories in patients with newly diagnosed with CKD G3-G5 according to EKFC eGFR that were not diagnosed according to CKD-EPI_2009_. The number of patients as well as the percentage of patients in each albuminuria category is shown.

Figure 4 shows the risk category distribution with percentages expressed over 100% of patients with albuminuria data for CKD-EPI_2009_ (Fig. 4a) or EKFC (Fig. 4b).

For CKD-EPI_2009_, 32.5% of participants with albuminuria data had CKD: 22.1% had G3–G5 CKD and 10.4% had G1–G2 CKD (3.3% G1 and 7.1% G2) (Fig. 4a). That is, ∼1 in 3 patients with CKD had early stage (G1–G2) CKD with relatively preserved kidney function.

For EKFC, 36.3% of participants with albuminuria data had CKD: 27.1% had G3–G5 CKD and 9.1% had G1–G2 CKD (2.0% G1 and 7.1% G2) (Fig. 4b). That is, ∼1 in 4 patients with CKD had CKD G1–G2.

The increase in the CKD G3–G5 population observed for EKFC when compared to CKD-EPI_2009_ was observed mainly in the A1 category (Fig. 4c): among the newly identified patients with potential CKD G3–G5 when using EKFC, 75% had A1 albuminuria and 25% A2–A3 albuminuria (Fig. 4d). Among patients with A2–A3 albuminuria, 422 were newly diagnosed as having CKD G3–G5 that were not diagnosed as having CKD G3–G5 using CKD-EPI_2009_, an increase of 12.4% (Fig. 4c and d).

### Impact on healthcare resources

**Figure 5:**
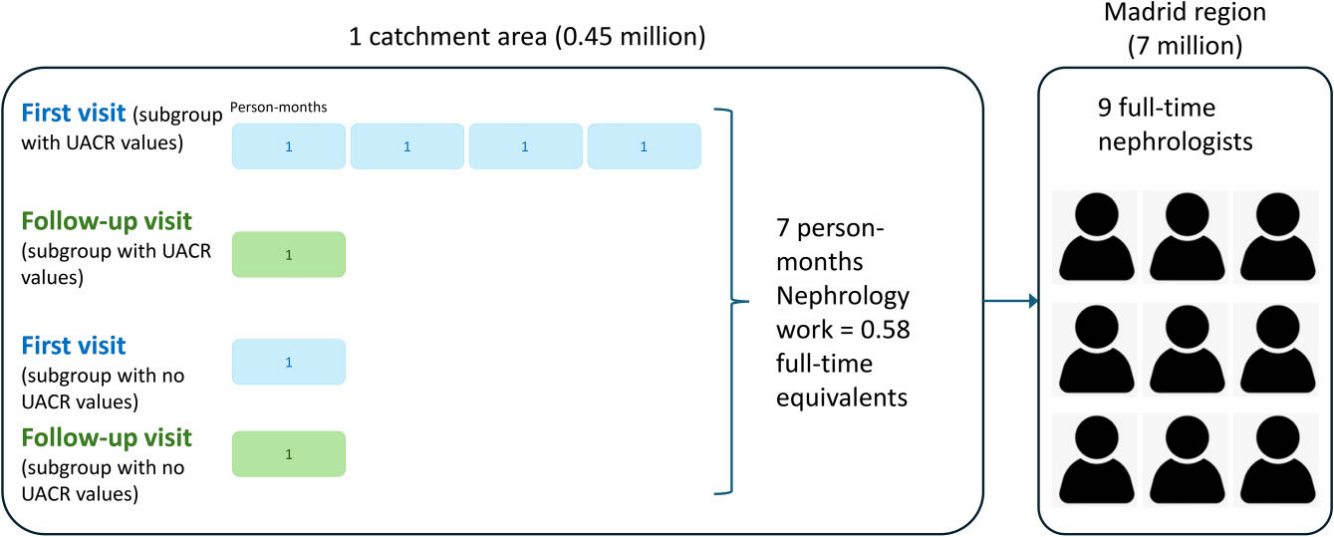
Impact of switching from CKD-EPI_2009_ to EKFC eGFR on nephrology workforce needs to provide guideline-recommended care for new patients diagnosed with CKD by the switch. Estimates were first generated for the first year in the catchment area under study and then extrapolated to the full region considering the different size of the population in each catchment area. Each rectangle represents 1 person-month of nephrology work. Nephrologist requirements to attend newly generated first visits are color-coded in blue and to attend follow-up visits in green. Calculations referred to the first 12 months after the switch.

As detailed in supplementary results, according to Spanish consensus guidance on care for patients with CKD [18] (Figs S5 and S6), switching from CKD-EPI_2009_ to EKFC may result in up to 7 person-months of new nephrology work, i.e. 0.58 full-time nephrologist equivalents, in the first year (Fig. 5) in one catchment area. In the region of Madrid [20], this would translate into roughly nine new full-time nephrologists (Fig. 5). Limiting nephrology care to those with CKD G4-G5 would decrease the number of needed nephrologists to 4 person-months, i.e. 0.33 full-time nephrologist equivalents for the catchment area that would translate into roughly five new full-time nephrologists in the Madrid region. Most (97%) new patients with CKD G4-G5 requiring nephrology care would be 75 years of age or older. Life expectancy at age 75 in Madrid is 15.5 years (i.e. up to age 90.5) (https://ec.europa.eu/eurostat/databrowser/view/demo_r_mlifexp__custom_17697972/default/table). If an age-adjusted definition of age-adjusted of low GFR was applied, these numbers would not change for people older than 65 years. However, ∼2000 people aged under 40 years would meet newly the low GFR definition for that age [19] in the catchment and, presumably, need healthcare evaluation. These people were not incorporated into the calculations of healthcare personnel needs, despite potentially representing >30 000 people region-wide.

## DISCUSSION

The main finding is that a switch from CKD-EPI_2009_ to EKFC may increase the prevalence of CKD, given the consistently lower eGFR values, especially in the population most at risk of CKD, the elderly. Although it is likely that the population with albuminuria available is biased toward higher risk of CKD, its analysis provides insight into the nature of the population that would be newly diagnosed as having CKD G3–G5 when switching from CKD-EPI_2009_ to EKFC. Most (75%) newly diagnosed CKD patients represented A1 albuminuria, a low-risk category. As a group, newly diagnosed CKD patients also had laboratory values consistent with milder CKD than those already diagnosed by CKD-EPI_2009_. However, the switch may still identify hundreds of new patients as having CKD that had higher risk (A2-A3 albuminuria) within a single catchment area. This will translate into thousands at the reginal level and tens of thousands in Europe. CKD in these people may have been missed until more advanced if UACR assessment is not comprehensive and there is evidence that it is not [21]. The increased CKD detection rate will likely increase referrals to nephrology initially, although, given the availability of multiple guideline-recommended cardiorenal protective drugs, it may also lead to lower long-term need for more advanced nephrology care.

Despite the shared pattern of decreasing eGFR with age, there were differences between equations. Higher eGFR values were observed with CKD-EPI_2009_ than with EKFC. On one hand, CKD-EPI_2009_ suggests loss of eGFR with age in people aged under 40 years but EKFC better estimates measured GFR in this age group [22]. On the other hand, CKD-EPI_2009_ also provides higher eGFR values in older people, although the line runs quite parallel to that of EKFC with increasing age.

When comparing the prevalence and risk categorization of patients with CKD G3–G5 using both formulas, there was a clear increase in prevalence with EKFC as compared to CKD-EPI_2009_. EKFC resulted in a reclassification of new patients into the G3 category, increasing G3 prevalence by 41.4%. Patients classified as CKD G3–G5 using CKD-EPI_2009_ had higher levels of markers of kidney function decline, as well as metabolic and inflammatory markers. This suggests that the health status of patients categorized by CKD-EPI_2009_ might be worse than those newly diagnosed by EKFC, who may benefit from earlier detection and treatment. A similar trend was observed in the CKD G3–G5 group in patients older than 65 years.

The higher prevalence of CKD when switching from CKD-EPI_2009_ to EKFC is open to two opposing interpretations, since EKFC may be identifying lower risk patients as having CKD.

On one hand, this may be considered overdiagnosis of CKD, especially by a vocal minority that disagrees with the current eGFR-based definition of CKD in the elderly [19], since new CKD G3-G5 diagnoses were mainly found among the elderly and a majority had A1 albuminuria and G3a. Additionally, there is a risk for public opinion to contest the diagnosis, given the current and evolving political and social environment regarding healthcare in general.

On the other, earlier identification may allow earlier intervention [13]. Current dual (SGLT2 inhibitor plus RAS inhibitor) or triple therapy [23] may be even safer than RAS monotherapy, on top of providing kidney and cardiovascular protection in different settings common in the elderly (diabetes, heart failure, CKD) and may event prevent CKD (SGLT2 inhibitors in type 2 diabetes mellitus) and heart failure [13, 23–25]. One in four newly diagnosed CKD G3-G5 patients also met albuminuria diagnostic thresholds to diagnose CKD. A low eGFR may trigger albuminuria testing for CKD risk stratification in people who otherwise may not have had access to albuminuria testing. The higher risk provided by the combination of low eGFR and high albuminuria would qualify patients for kidney and cardiovascular protective therapy, consisting of up to four drugs to decrease cardiorenal risk, according to 2024 KDIGO CKD guidelines [4]. In this regard, the race-free CKD-EPI_2021_ equation underestimates GFR in African Americans and this is considered an advantage because it allows earlier intervention [8]. In summary, a lower eGFR may fit well with earlier intervention and the novel concepts of primary prevention of CKD: people who have CKD G3–G5 by EKFC and not by CKD-EPI_2009_ may become candidates for primary prevention aimed at preventing CKD as defined by CKD-EPI_2009_ [13, 24]. In many European and global settings, uptake of albuminuria testing is suboptimal, even among people with diabetes mellitus who have much higher uptake than other risk groups such as people with hypertension [26, 27]. In >75% of people initiating KRT in Europe, the cause is not diabetes mellitus [28]. Thus, in environments where albuminuria uptake is low, CKD-EPI_2009_ may not identify as having CKD those patients with A2–A3 albuminuria because of lack of testing. Being identified as having G3-G5 by EKFC may potentially trigger albuminuria assessment and initiation of therapy for CKD. As there was likely a reason why 15% of patients had albuminuria data, over 400 patients with A2–A3 albuminuria may be an overrepresentation. Still, a number in the hundreds is significant in a catchment area that generates ∼50 to 60 new kidney failure patients requiring KRT per year [28, 29]. Unlike UACR, eGFR is widely available and may represent the port of entry for CKD evaluation in healthcare systems where UACR is not reimbursed outside diabetes (e.g. hypertension). The precise numbers may, however, be lower outside these high-risk populations.

Age-adapted eGFR thresholds for low eGFR have been advocated to diagnose CKD [19], which differ for the consensus CKD definition. The use of such thresholds would add newly diagnosed younger (<40 years) individuals and remove older (>65 years) individuals, resulting in a net decrease of people newly diagnosed of CKD. However, these changes would not modify the need for additional nephrology workforce.

Our study expands recent Dutch observations [30] to the region with longest life expectancy in Europe, located in Southern Europe, and to a different healthcare setting (combined hospital and primary care vs hospital). Furthermore, we also assessed the impact on nephrology referral and the increased need for nephrologists. We observed a larger increase in CKD prevalence on switching equations (40% vs 29%), likely resulting from the older age in our participants (median age 56 vs. 51 years). Data were also aligned with recent observations in a pan-European healthy cohort, as were the different shapes of age-associated differences between EKFC and CKD-EPI_2009_ for people younger than 40 years [17]. Despite being healthcare users, >50% in our study did not meet eGFR thresholds to diagnose CKD until age 91+ (CKD-EPI_2009_) or 82+ years (EKFC), implying that most older individuals remain with eGFR values above the threshold to diagnose CKD.

Some limitations should be acknowledged. Only healthcare users were studied, so it may be biased against healthier people and CKD prevalence may be overestimated. This may be magnified by the use of a single determination to presumptively diagnose CKD. However, both biases will affect any eGFR equation, and this study would still be valid to compare eGFR equations. These equations were developed in populations that did not include centenarians. The European published *Q* value was used [15], which was calculated in Belgium and Sweden. However, this does not detract from the key message that for the same value of serum creatinine, EKFC eGFR values were lower than those obtained using CKD-EPI_2009_, potentially leading to a new diagnosis of CKD. Different healthcare systems may have different rules for care by primary care or nephrology, thus, the healthcare resources impact should be analyzed for different healthcare systems with different demographic pyramids and life expectancies. Patients who are not expected to be under nephrology care (as per guidelines for nephrology referral), may be under nephrology care already for other reasons. Finally, this study does not assess whether those reclassified are at increased risk for adverse kidney-related outcomes, and thus whether they would meaningfully benefit from earlier diagnosis. Extrapolation to the full Madrid region is approximate and has not used detailed demographic data for the whole region. All laboratory assessments have limitations and those of albuminuria and creatinine are extensively discussed in clinical guidelines, including the potential for menstruation to increase albuminuria [4].

Among the strengths, the study involved a large, contemporary population with a wide age range drawn from both hospital and primary care in an environment where the very elderly are more common than in other European regions and receive the full attention of the healthcare system given their longer life expectancy.

In conclusion, a move from the creatinine-based CKD-EPI_2009_ to the EKFC eGFR equation would increase CKD prevalence, especially among the elderly and people with A1 albuminuria. However, it may also identify people with “occult” A2–A3 albuminuria who may have been missing a CKD diagnosis in healthcare systems that do not systematically assess albuminuria. Future studies are needed to define whether patients switching from no-CKD to CKD G3–G5 status are at similar or higher risk for CKD-associated adverse events (kidney failure, all-cause mortality, acute kidney injury, cardiovascular disease) than those not switched or those originally diagnosed of CKD G3–G5 and whether medical intervention as result of the switch improves outcomes. These findings may inform future decisions about which eGFR equation to adopt and support expanding the population for whom albuminuria testing is recommended [31].

## Supplementary Material

sfaf278_Supplemental_Files

## Data Availability

The data underlying this article will be shared on reasonable request to the corresponding author.
