# Supplementary material for: Impact of switching from the CKD-EPI2009 to the EKFC equation on the epidemiology of chronic kidney disease and nephrology workload
Source: Clin Kidney J. 2025 Sep 8;18(10):sfaf278. doi: 10.1093/ckj/sfaf278 (PMC12554879; doi:10.1093/ckj/sfaf278)
Supplement: sfaf278_Supplemental_Files [file sfaf278_supplemental_files.zip › CKD epidemiology suppl files.pdf]

Supplementary data

Supplementary methods

Supplementary results

Supplementary tables

Supplementary figure legends

Supplementary references

Supplementary methods

GFR equations

Both CKD-EPI$_{2009}$ and EKFC are serum creatinine-based equations that have been externally validated and are recommended by the latest KDGIO CKD guidelines, which extensively discuss both (1). A key difference is the inclusion in EKFC of a Q value, which should be assessed for target population and consist of the normal level of creatinine in that region. Additionally, the specific EKFC equation differs for people older and younger than 40 years. As a consequence, eGFR in healthy populations is stable from age 18 to 40 years as assessed by EKFC but continually decreases when assessed by CKD-EPI2009 (2,3).
The equations were applied as follows:

	**Age**	**Sex**	**Serum creatinine (mg/dL)**	**Equation**
CKD-EPI 2009	≥18	Female	SCr ≤ 0.7	$144 \times (SCr/0.7)^{-0.329} \times (0.993)^{Age}$
			SCr > 0.7	$144 \times (SCr/0.7)^{-1.209} \times (0.993)^{Age}$
		Male	SCr ≤ 0.9	$141 \times (SCr/0.9)^{-0.411} \times (0.993)^{Age}$
			SCr > 0.9	$141 \times (SCr/0.9)^{-1.209} \times (0.993)^{Age}$
EKFC	18-40	Female	SCr/Q < 1.0	$107.3 \text{ x } (SCr/Q)^{-0.322}$
			SCr/Q ≥ 1.0	$107.3 \text{ x } (SCr/Q)^{-1.132}$
		Male	SCr/Q < 1.0	$107.3 \text{ x } (SCr/Q)^{-0.322}$
			SCr/Q ≥ 1.0	$107.3 \text{ x } (SCr/Q)^{-1.132}$
	>40	Female	SCr/Q < 1.0	$107.3 \text{ x } (SCr/Q)^{-0.322} \text{ x } 0.990^{(Age-40)}$
			SCr/Q ≥ 1.0	$107.3 \text{ x } (SCr/Q)^{-1.132} \text{ x } 0.990^{(Age-40)}$
		Male	SCr/Q < 1.0	$107.3 \text{ x } (SCr/Q)^{-0.322} \text{ x } 0.990^{(Age-40)}$
			SCr/Q ≥ 1.0	$107.3 \text{ x } (SCr/Q)^{-1.132} \text{ x } 0.990^{(Age-40)}$

Estimation of Nephrology workload

The Primary Care workload for newly diagnosed CKD patients was not estimated because all participants were healthcare users in the public health systems and, thus, were already followed by Primary Care. The Nephrology workload was estimated using the 2022 Spanish consensus guidance documents for Nephrology referral based on the KDIGO risk heatmap (4). The number of patients who may require Nephrology consultation because they have CKD A3 OR G3bA1 OR G3A2, OR G4 OR G5 was estimated. The new need for Nephrology care was estimated based on the switch from risk categories managed by Primary Care to risk categories managed by Nephrology (red cells) or that may require (pink cells) Nephrology consultation (**Figure S5**). This was a conservative estimate in which the first Nephrology visit was accounted for, but only 1 additional follow-up visit was considered for risk categories followed by Nephrology. The work schedule of Nephrology was estimated at 7.5 h/day, 5 days per week. A first Nephrology consultation would take 30 min for a total of 13 visits per day, accounting for other clinical, educational and administrative activities. Repeat Nephrology visits were considered to take 20 minutes, for a maximum of 18 per day. Each month was considered to contain 17 working days, after removal of weekends (104 days), festivities (12 days per year in Madrid), vacation (22 days per year), free disposal (6 per years) and days allowed for course and congresses (15 per year), all according to the work conditions in the Regional healthcare system. Additionally, the number of working days was also estimated for older workers that have 7 additional non-working days per year.
In a sensitivity analysis that would underestimate the need for nephrologists, the number of nephrologists needed to care for newly diagnosed patients with CKD A3 OR G4 OR G5 was estimated (i.e. red cells in **Figure S5**). The difference between both equations was limited to people newly diagnosed with CKD G4-G5.

Supplementary results

Laboratory characteristics of patients with CKD G3-G5

Table 2 presents key laboratory data for patients with CKD G3-G5 categorized using CKD-EPI$_{2009}$ or EKFC.

Patients classified as CKD G3-G5 based on CKD-EPI$_{2009}$ were less frequently women and had higher rates of diabetes and specialty care compared to those with EKFC G3-G5. Metabolic and inflammatory markers, including glucose, uric acid, triglycerides, HbA1c, total leukocyte count and ferritin levels, as well as kidney and injury function markers such as creatinine, urea and UACR were higher for CKD-EPI$_{2009}$ G3-G5 than for EKFC G3-G5.

Patients aged >65 years with CKD G3-G5 diagnosed using CKD-EPI$_{2009}$ were older and had higher rates of diabetes and specialty care (18.4% vs. 16.6%) than those with CKD G3-G5 using CKD-EPI$_{2009}$ (**Table S3**). Inflammatory and kidney function and injury markers (serum creatinine, UACR, urinary protein, PTH, HbA1c, potassium, leukocyte count, ferritin) were again higher among CKD G3-G5 CKD-EPI$_{2009}$ than among CKD G3-G5 EKFC patients.

Impact on healthcare resources

This was a cohort of healthcare users. Thus, all of them were followed by Primary Care, although some had been also evaluated or were being followed by Specialty Care. Spanish consensus guidance documents establish rules for Nephrology consultations based on the KDIGO risk heatmap (1). The new need for Nephrology care was estimated based on the switch from risk categories managed by Primary Care to risk categories managed by Nephrology or that may require Nephrology consultation (**Figure S5**).

Since risk categories depend on both eGFR and UACR, we first estimated the need for new Nephrology care in the 33,789 patients who had albuminuria data. The number of patients who may require Nephrology consultation because they have CKD G3bA1 OR G3A2, OR G4 OR G5 OR A3 would increase from 5239 (CKD-EPI$_{2009}$) to 6132 (EKFC); i.e., 893 (+17.0%) additional patients would be diagnosed as having CKD, the difference corresponding to patients switching from one G category to the next G category. A new diagnosis of CKD requiring referral to Nephrology (red cells in **Figure S5**) or justifying referral to Nephrology (pink cells in **Figure S5**). The default scenario estimated a maximum of 893 novel CKD diagnosis that justified or required referred to Nephrology, generating 893 first Nephrology visits, i.e., 69 full-time days of Nephrology work, roughly 4 person-months (**Figure 5, S6A**). This is considered the most likely scenario, given the work overload of Primary care physicians.

In addition, the number of people requiring Nephrology follow-up would increase from 2029 to 2267 (+11.7%). This would mean 238 new patients to follow, requiring at least 13 full-time days of Nephrology work for one follow-up visit, although these patients may need more than one visit per year. This may amount to roughly 0.8 person-month (**Figure 5, S6A**). This need is likely to be recurrent.

Overall, the 33,789 patients who had albuminuria data would generate roughly 5 persons-months of Nephrology work (**Figure 5**). However, they represented only 15% of the full population.

Next, we estimated Nephrology care for patients in the full cohort who would be diagnosed of CKD G4-G5 using EKFC but not with CKD-EPI$_{2009}$. They would increase from 1939 to 2483 (+28%), i.e., 544 newly diagnosed patients with CKD G4-G5 that would require first evaluation and follow-up. Of them, 274 would correspond to patients having albuminuria data. Thus, they would have already contributed to the calculation made above. Thus, 270 additional patients would need a first Nephrology visit and also follow-up. The first Nephrology visits would need 21 days of Nephrology work, i.e., roughly 1.3 person-month (**Figure 5**). A single follow-up visit would entail 15 full-time days, another 0.9 person-month. Overall, the new CKD G4-G5 patients among those not having UACR data would add roughly another 2.2 person-months of Nephrology work (**Figure 5**).

Combining both estimates would mean a total of 7 person-months of Nephrology work, i.e. 0.58 full-time equivalents in the first year (**Figure 5**) as a rough estimate of the impact of a switch from CKD-EPI$_{2009}$ to EKFC on Nephrology work in this specific catchment area, only part of which may not be recurrent in subsequent years. In the region of Madrid, which has 7 million inhabitants, these needs would translate into roughly 9 new full-time nephrologists (**Figure 5**).

In a sensitivity analysis, the number of nephrologists needed to care for newly diagnosed patients with CKD A3 OR G4 OR G5 was estimated (**Figure S6B**) at 4 person-months, i.e., 0.33 full-time nephrologist equivalents for the catchment area that would translate into roughly 5 new full-time nephrologists in the region of Madrid.

Supplementary tables

Table S1. Missing values, n (%)

Variable	Missing values, n (%)
Age (years)	0 (0%)
Glucose (mg/dl)	1844 (1%)
UACR (mg/g)	182830 (84%)
HbA1c (%)	133944 (62%)

Table S2a. Clinical and laboratory values of patients with CKD G3 according to-EPI2009 and EKFC				
**Variable**	**G3 CKD-EPI$_{2009}$ (n=18 907)**	**G3 EKFC (n=26 657)**	**p value**	**G3 EKFC but not G3 with CKD-EPI$_{2009}$ (n=8 344)**
**Age (years)**	80 (72-87)	79 (72-86)	<0.05	79 (72-85)
**Women, n (%)**	11054 (59%)	16101 (60%)	NS	5459 (65%)
**Diabetes, n (%)**	4212 (22%)	5370 (20%)	NS	1324 (16%)
**Hospital, n (%)**	3074 (16%)	3936 (15%)	NS	1003 (12%)
**Glucose (mg/dl)**	100.00 (90.00-114.00)	99.00 (90.00-113.00)	<0.05	98.00 (89.00-110.00)
**HbA1C**	5.70 (5.40-6.20)	5.70 (5.40-6.20)	<0.05	5.70 (5.40-6.10)
**Creatinine (mg/dl)**	1.19 (1.02-1.36)	1.09 (0.94-1.27)	<0.05	0.91 (0.85-1.08)
**eGFR CKD-EPI$_{2009}$ (ml/min/1.73m^2)**	51.3 0(44.10-56.30)			63.03 (61.54-64.98)
**eGFR EKFC (ml/min/1.73m^2)**		51.20 (44.10-56.10)		57.49 (55.84-58.85)
**UACR (mg/g)**	20.70 (8.60-65.80)	17.60 (7.80-54.60)	<0.05	11.90 (6.40-30.17)

Table S2b. Clinical and laboratory values of patients with CKD G4 according to-EPI2009 and EKFC				
**Variable**	**G4 CKD-EPI$_{2009}$ (n=1523)**	**G4 EKFC (n=2049)**	**p value**	**CKD G4 EKFC but not CKD-EPI$_{2009}$ (n=553)**
**Age (years)**	85 (77-91)	87 (79-92)	<0.05	89 (84-93)
**Women, n (%)**	885 (58%)	1274 (62%)	NS	407 (74%)
**Diabetes, n (%)**	555 (36%)	715 (35%)	NS	169 (31%)
**Hospital, n (%)**	487 (32%)	619 (30%)	NS	141 (25%)
**Glucose (mg/dl)**	102.0 (90.0-125.0)	102.0 (90.0-123.0)	<0.05	101.00 (90.00.120.00)
**HbA1C**	5.90 (5.50-6.80)	5.90 (5.50-17.00)	0.43	5.90 (4.20-6.60)
**Creatinine (mg/dl)**	2.05 (1.76-2.43)	1.89 (1.59-2.24)	<0.05	1.49 (1.42-1.74)
**eGFR CKD-EPI$_{2009}$ (ml/min/1.73m^2)**	25.00 (21.60-27.80)			28.88 (28.16-29.44)
**eGFR EKFC (ml/min/1.73m^2)**		25.40 (22.00-28.00)		31.51 (30.75-32.37)
**UACR (mg/g)**	92.50 (25.70-427.10)	70.40 (22.20-324.70)	0.29	34.50 (14.35-108.45)

Table S2c. Clinical and laboratory values of patients with CKD G5 according to-EPI2009 and EKFC				
**Variable**	**G5 CKD-EPI$_{2009}$ (n=416)**	**G5 EKFC (n=434)**	**p value**	**CKD G5 EKFC but not CKD-EPI$_{2009}$ (n=25)**
**Age (years)**	74 (61-85)	76 (62-86.8)	<0.05	91 (88-95)
**Women, n (%)**	186 (45%)	201 (46%)	NS	18 (72%)
**Diabetes, n (%)**	138 (33%)	144 (33%)	NS	8 (32%)
**Hospital, n (%)**	190 (46%)	196 (45%)	NS	8 (32%)
**Glucose (mg/dl)**	103 (89-134)	102 (89-134)	<0.05	97.00 (89.00-122.00)
**HbA1C**	5.8 (5.3-6.7)	5.8 (5.3-6.7)	0.81	5.70 (5.20-6.40)
**Creatinine (mg/dl)**	5.55 (4.0-7.79)	5.42 (3.8-7.7)	0.38	2.74 (2.54-3.22)
**eGFR CKD-EPI$_{2009}$ (ml/min/1.73m^2)**	8.1 (5.9-11.9)			15.39 (15.15-15.59)
**eGFR EKFC (ml/min/1.73m^2)**		8.9 (6.4-12.5)		14.52 (14.35-14.80)
**UACR (mg/g)**	521 (139-1333)	444.1 (105-1273.1)	0.52	112.15 (46.68-296.60)

**Table S3. Clinical and laboratory values of patients with CKD G3-G5 older than 65 years according to-EPI2009 and EKFC**				
**Variable**	**CKD G3-G5 EPI2009** (n=17939)	**CKD G3-G5 EKFC** (n=25588)	**P value**	**CKD G3-G5 EKFC but not CKD-EPI2009** (n=7649)
Age (years)	82.0 (76.0-88.0)	81.0 (75.0-88.0)	<0.0001	80.0 (74.0-86.0)
Women, n (%)	10824 (60.3%)	15838 (61.9%)	0.001	5014 (65.6%)
Diabetes, n (%)	4398 (25.6%)	5642 (23.1%)	<0.0001	1244 (17.2%)
Specialty care, n (%)	3301 (18.4%)	4249 (16.6%)	<0.0001	948 (12.4%)
Glucose (mg/dl)	100.0 (90.0-116.0)	99.0 (90.0-114.0)	<0.0001	98.0 (89.0-110.0)
Creatinine (mg/dl)	1.2 (1.0-1.4)	1.1 (0.9-1.3)	<0.0001	0.9 (0.8-1.0)
eGFR CKD-EPI2009 (ml/min/1.73m^2)	49.5 (40.4-55.4)	54.7 (45.0-61.1)	<0.0001	63.3 (61.6-65.1)
eGFR EKFC (ml/min/1.73m^2)	44.8 (36.8-50.1)	49.4 (40.8-55.2)	<0.0001	57.2 (55.6-58.6)
UACR (mg/g)	24.8 (9.8-84.7)	21.5 (8.8-70.8)	0.006	12.2 (6.5-30.9)
HbA1c (%)	5.8 (5.4-6.4)	5.7 (5.4-6.3)	<0.0001	5.7 (5.4-6.1)

Table S4. Clinical and laboratory values of patients with CKD G3a older than 65 years according to-EPI2009 and EKFC			
**Variable**	**CKD G3a EPI2009** (n=11544)	**CKD G3a EKFC** (n=16483)	**P value**
Age (years)	80.6 (74.0-87.0)	79.0 (74.0-85.0)	<0.0001
Women, n (%)	6953 (60.2%)	10102 (61.3%)	0.074
Diabetes, n (%)	2389 (21.7%)	3035 (19.4%)	<0.0001
Specialty care, n (%)	1690 (14.6%)	2166 (13.1%)	<0.0001
Glucose (mg/dl)	99.0 (90.0-113.0)	99.0 (90.0-112.0)	ns
Creatinine (mg/dl)	1.07 (0.97-1.23)	0.98.(0.89-1.15)	<0.0001
eGFR CKD-EPI2009 (ml/min/1.73m^2)	54.0 (50.0-57.2)	59.5 (55.2-63.0)	<0.0001
eGFR EKFC (ml/min/1.73m^2)	48.8 (45.2-51.8)	53.7 (49.9-57.2)	<0.0001
UACR (mg/g)	16.7 (7.6-47.3)	13.9 (6.8-36.8)	0.006
HbA1c (%)	5.7 (5.4-6.2)	5.7 (5.4-6.2)	ns

Table S5. Clinical and laboratory values of the study population with UACR values			
**Variable**	**All** (n=33 789)	**Men** (n=17224)	**Women** (n=16 565)
Age (years)	65 (52-76)	64 (53-75)	65 (50-78)
Women, n (%)	16 565 (49%)	--	--
Hypertension, n (%)	15 499 (45.8%)	8 455 (53%)	7 041 (45%)
Diabetes, n (%)	9 276 (27.4%)	5 363 (34%)	3 910 (25%)
Specialty care, n (%)	3410 (10.1%)	1850 (11%)	1560 (9%)
CKD G3-G5<br>According to CKD-EPI$_{2009}$, n (%)<br>According to EKFC, n (%)	 7 478 (22.1%)<br>9 165 (27.1%)	 3 876 (22.5%)<br>4 645 (27%)	 3 602 (22%)<br>4 520 (27%)
Glucose (mg/dl)	99 (88-116)	101 (91-121)	96 (87-111)
Creatinine (mg/dl)	0.89 (0.75-1.09)	1.00 (0.88-1.19)	0.77 (0.67-0.91)
eGFR CKD-EPI$_{2009}$ (ml/min/1.73m^2)	81.51 (63.00-94.92)	80.30 (62.45-93.22)	82.75 (63.55-96.77)
eGFR EKFC (ml/min/1.73m^2)	75.41(58.09-89.62)	75.17 (58.34-88.50)	75.77 (57.84-90.96)
UACR (mg/g)	8.30 (4.40-22.80)	7.80 (4.00-25.50)	8.70 (4.90-20.80)
HbA1c (%)	5.70 (5.30-6.50)	5.80 (5.40-6.60)	5.70 (5.30-6.30)

Supplementary figure legends

Figure S1. Age distribution. There were 199 patients aged over 99 years.

Figure S2. Median eGFR values according to age for CKD-EPI$_{2009}$. and EKFC. A) Men. **B)** Women. The horizontal discontinuous orange line marks 60 ml/min/1.73 m^2, the threshold to diagnose CKD based on eGFR values.

Figure S3. Prevalence of KDIGO G categories for eGFR values obtained using CKD-EPI$_{2009}$. and EKFC, expressed as percentage of the full population. A) Men. **B)** Women.

Figure S4. KDIGO G risk categories and prevalence of CKD G3-G5 in the full study population according to sex and age. A) All, CKD-EPI$_{2009}$. **B)** All, EKFC. **C)** Men, CKD-EPI$_{2009}$. **D)** Men, EKFC. **E)** Women, CKD-EPI$_{2009}$. **F)** Women, EKFC.

Figure S5. Owner of care for patients with CKD according to the 2022 consensus documents published by Nephrology and Primary Care Scientific Societies in Spain. (4).

Figure S6. Estimation of nephrologist needs to attend newly diagnosed CKD patients during the first year after the switch from CKD-EPI$_{2009}$ to EKFC. The number of new patients with CKD was estimated and transformed into hours needed to be attended as first (blue) or follow-up (green) visits, based on national guidelines summarized in figure S5. These were transformed into number of days needed and the number of days, into the number of person-months as detailed in the text. As the number of working days per year differs for younger and older nephrologists, two estimates were made for each condition. **A)** Patients for whom Nephrology visits should be considered (G3a3, G3bA1, G3bA2) and patients who require Nephrology follow-up (A3, G4, G5) were analyzed. **B)** Only patients who required Nephrology follow-up were analyzed.

Supplementary references

1. Kidney Disease: Improving Global Outcomes (KDIGO) CKD Work Group. KDIGO 2024 Clinical Practice Guideline for the Evaluation and Management of Chronic Kidney Disease. Kidney Int. 2024 Apr;105(4S):S117-S314
2. Astley ME, Chesnaye NC, Gambaro G, et al. Prevalence of reduced eGFR in European adults using KDIGO and age-adapted eGFR thresholds. Nephrol Dial Transplant. 2025 Jul 17:gfaf112. doi: 10.1093/ndt/gfaf112.
3. Astley ME, Chesnaye NC, Hallan S, et al. Age- and sex-specific reference values of estimated glomerular filtration rate for European adults. Kidney Int. 2025;107(6):1076-1087.
4. García-Maset R, Bover J, Segura de la Morena J, et al. Information and consensus document for the detection and management of chronic kidney disease. Nefrologia (Engl Ed). 2022;42(3):233-264
