# Supplementary figures and images for: Impact of switching from the CKD-EPI2009 to the EKFC equation on the epidemiology of chronic kidney disease and nephrology workload
Source: Clin Kidney J. 2025 Sep 8;18(10):sfaf278. doi: 10.1093/ckj/sfaf278 (PMC12554879; doi:10.1093/ckj/sfaf278)

## Slide 1
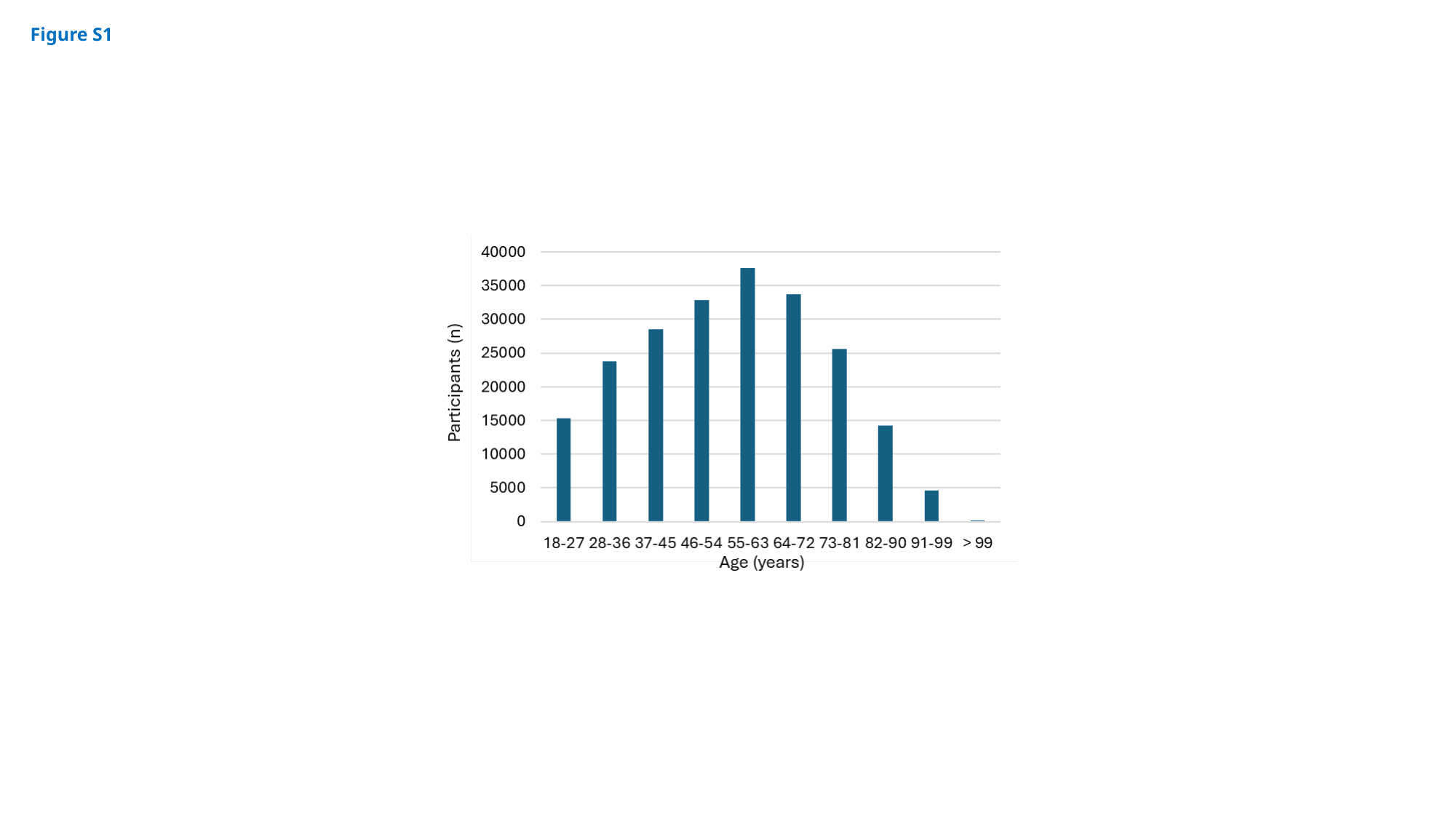

Figure S1

Supplement: sfaf278_Supplemental_Files [file sfaf278_supplemental_files.zip › Figure S1 nm R1.pptx]

## Slide 1
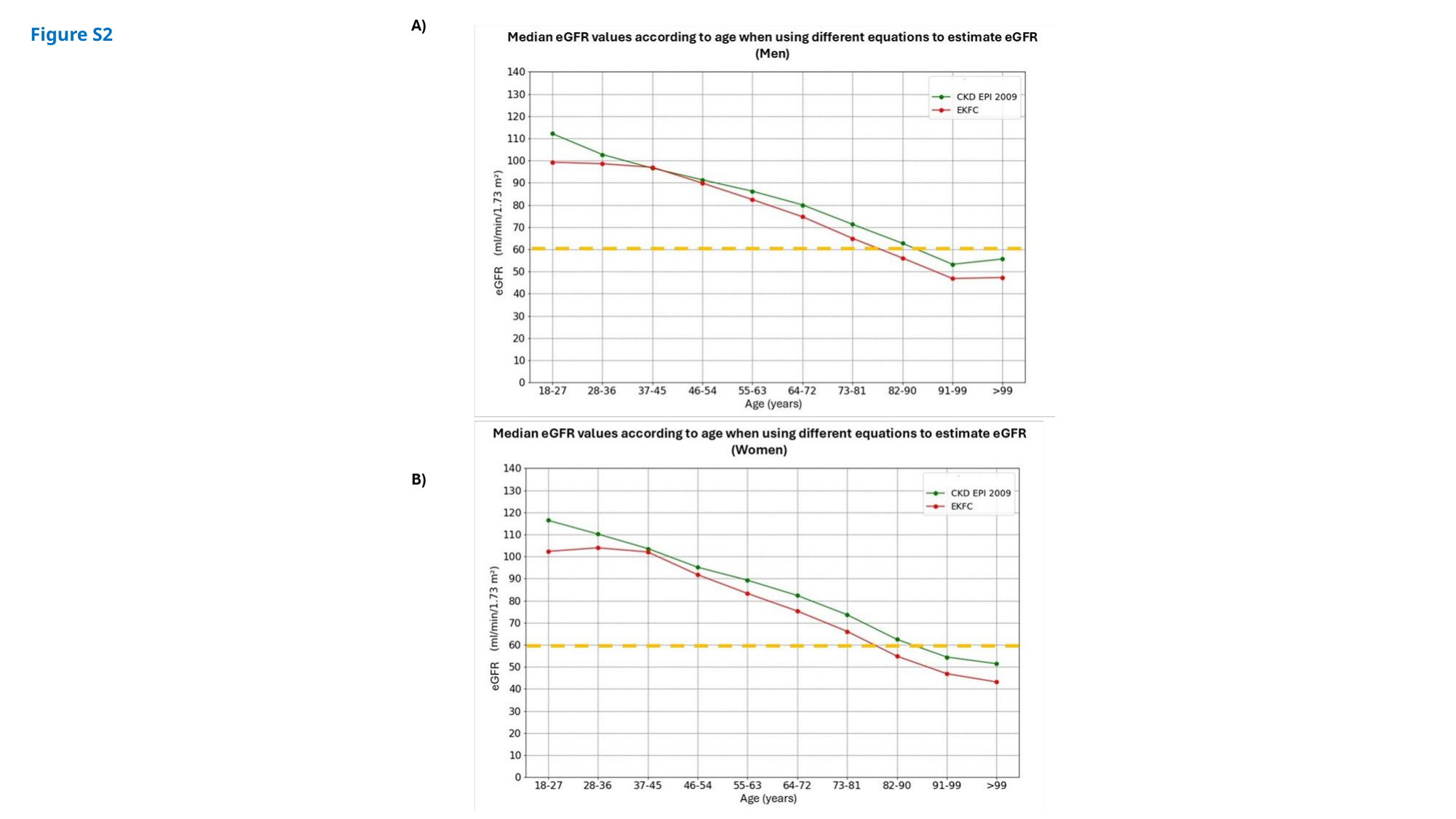

Figure S2

Supplement: sfaf278_Supplemental_Files [file sfaf278_supplemental_files.zip › Figure S2 nm R1.pptx]

## Slide 1
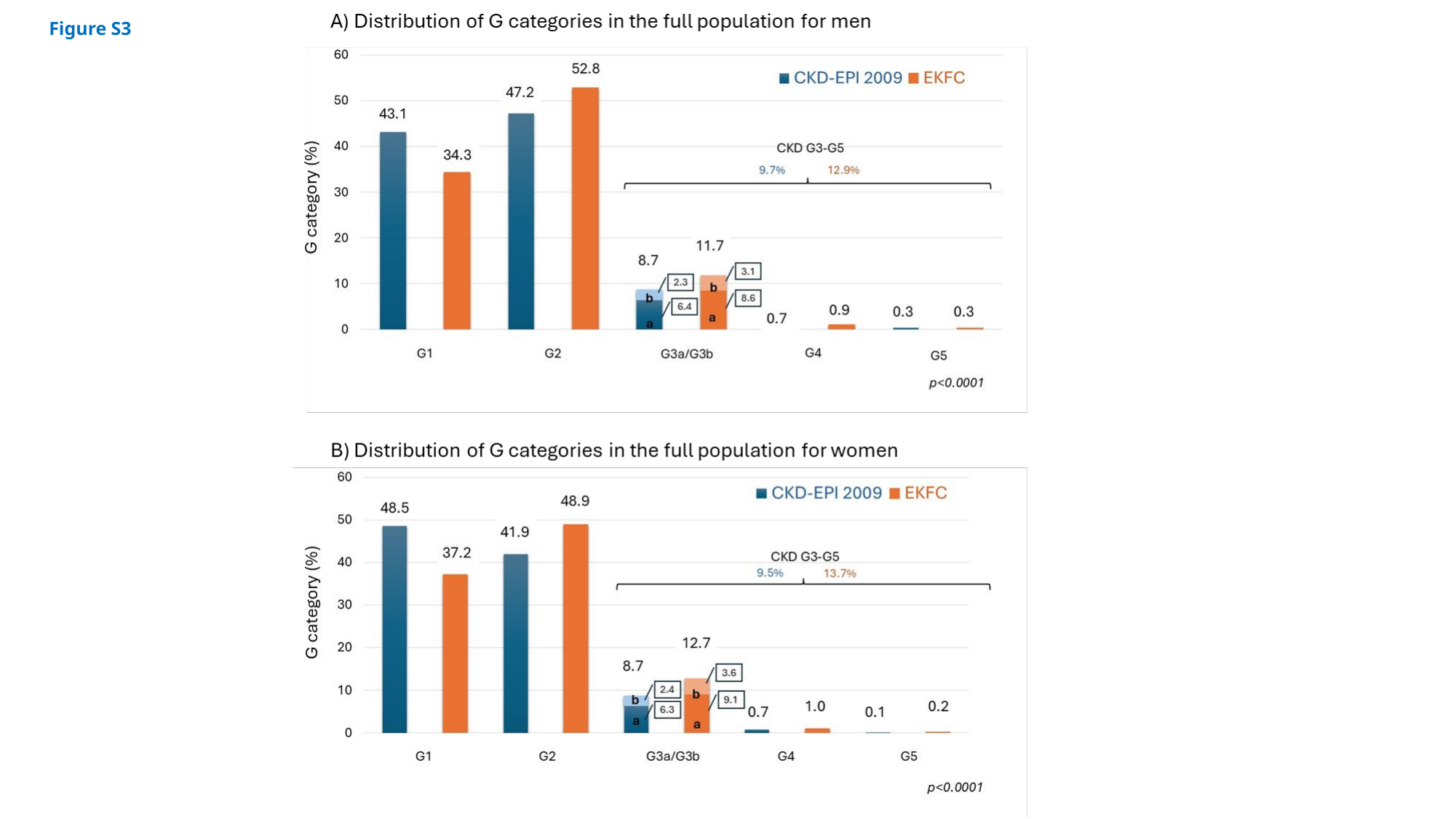

Figure S3

Supplement: sfaf278_Supplemental_Files [file sfaf278_supplemental_files.zip › Figure S3 nm R1.pptx]

## Slide 1
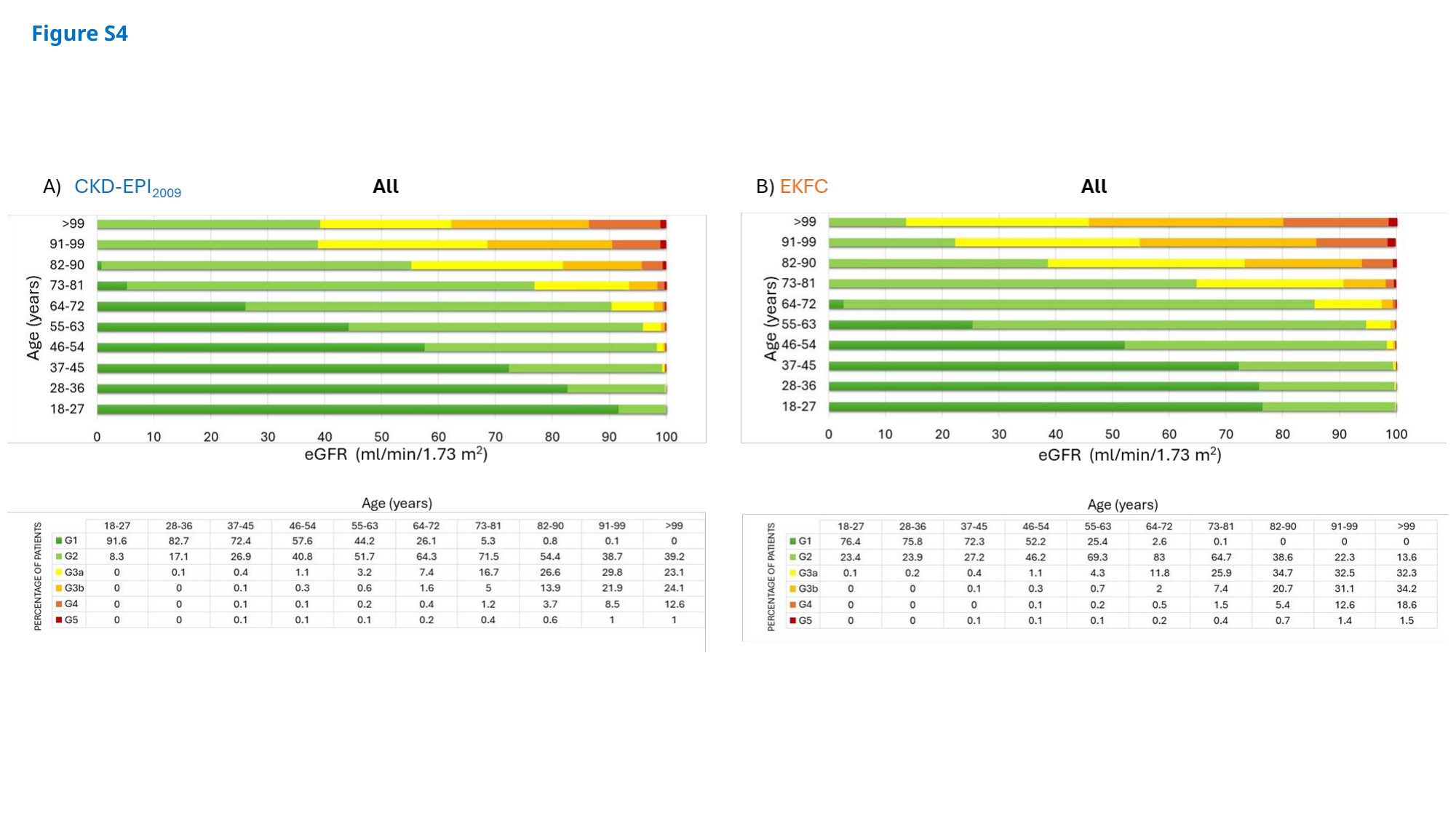

Figure S4

## Slide 2
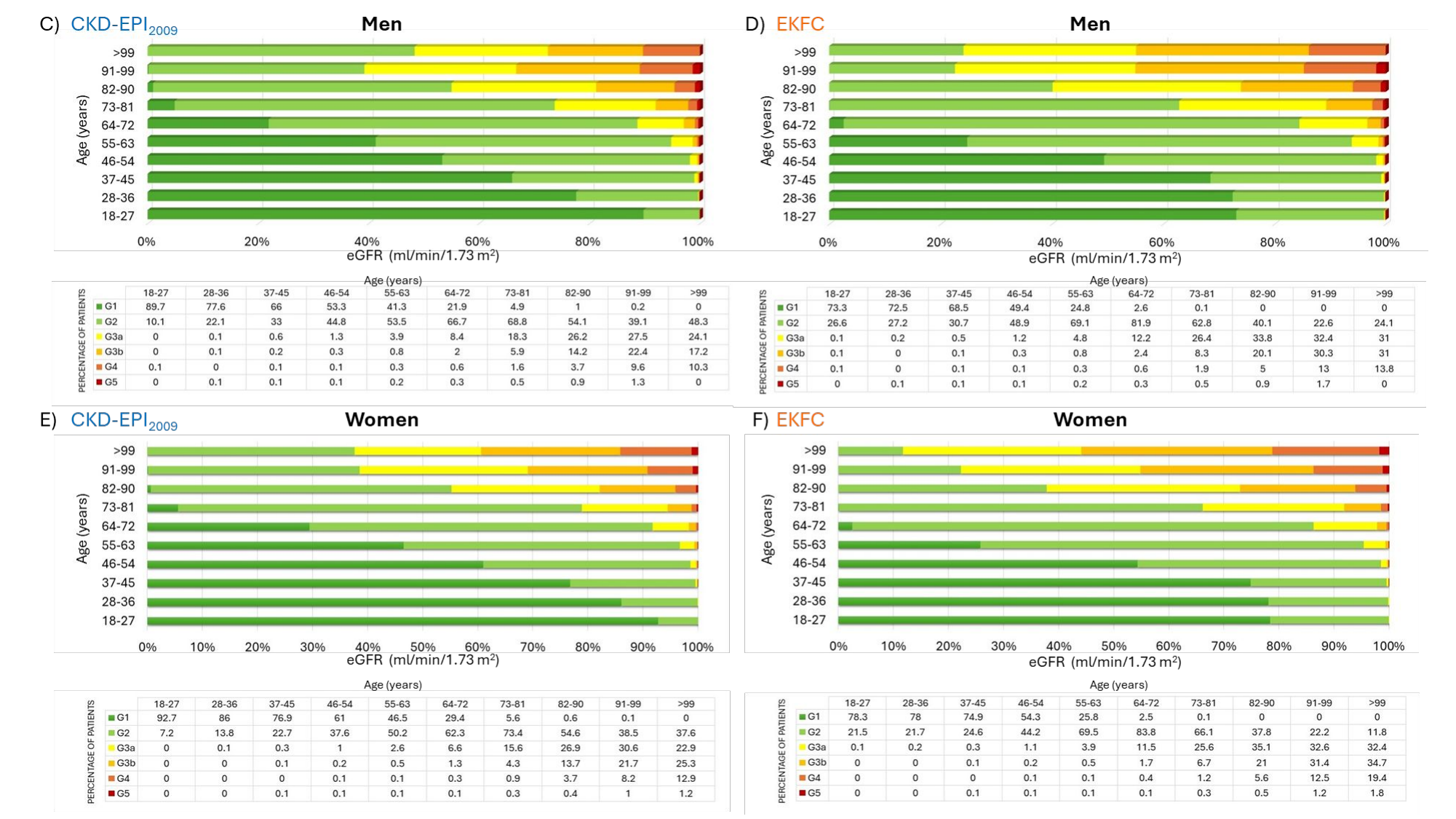

Supplement: sfaf278_Supplemental_Files [file sfaf278_supplemental_files.zip › Figure S4 nm R1.pptx]

## Slide 1
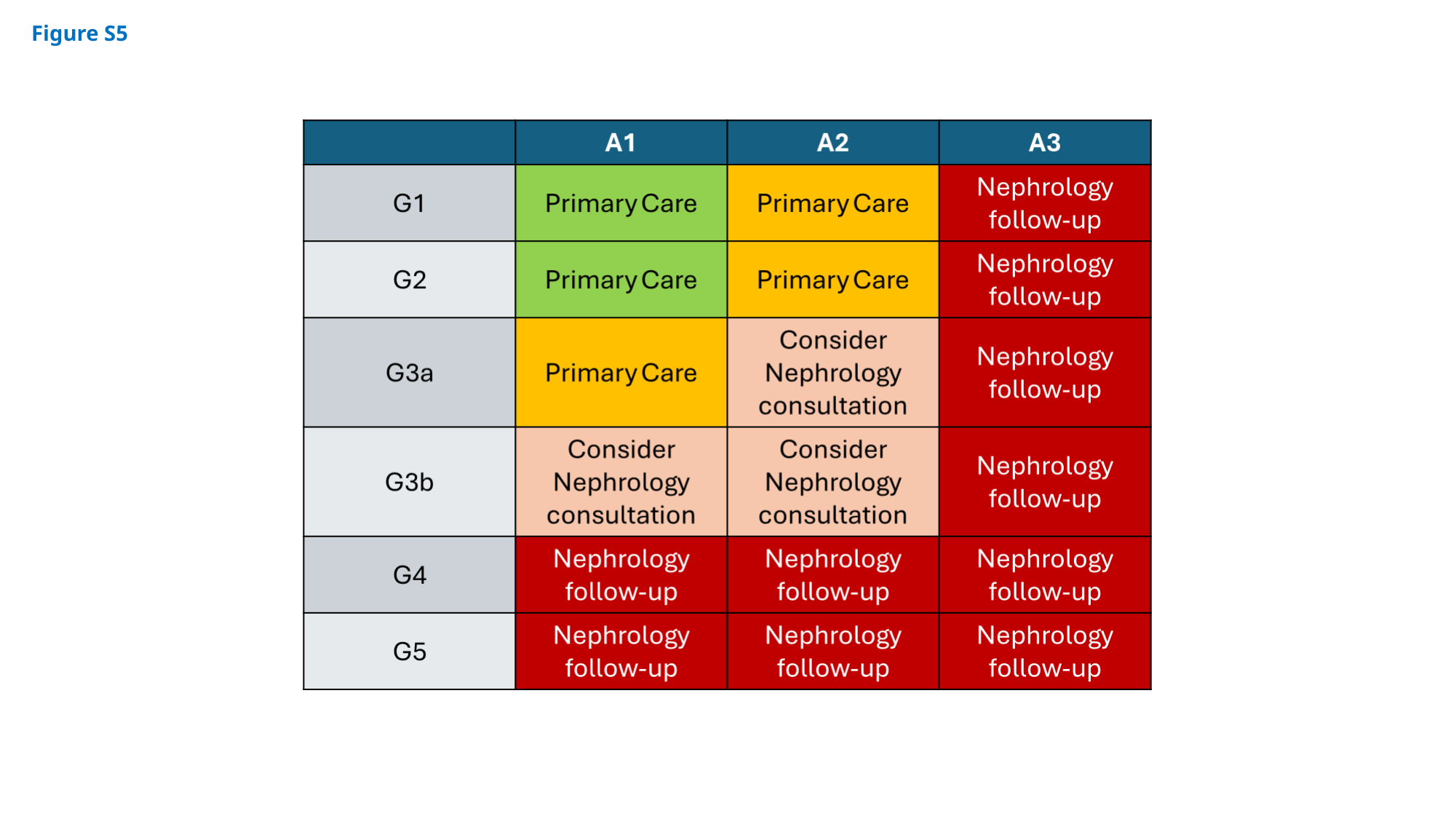

Figure S5

Supplement: sfaf278_Supplemental_Files [file sfaf278_supplemental_files.zip › Figure S5 bn R1.pptx]

## Slide 1
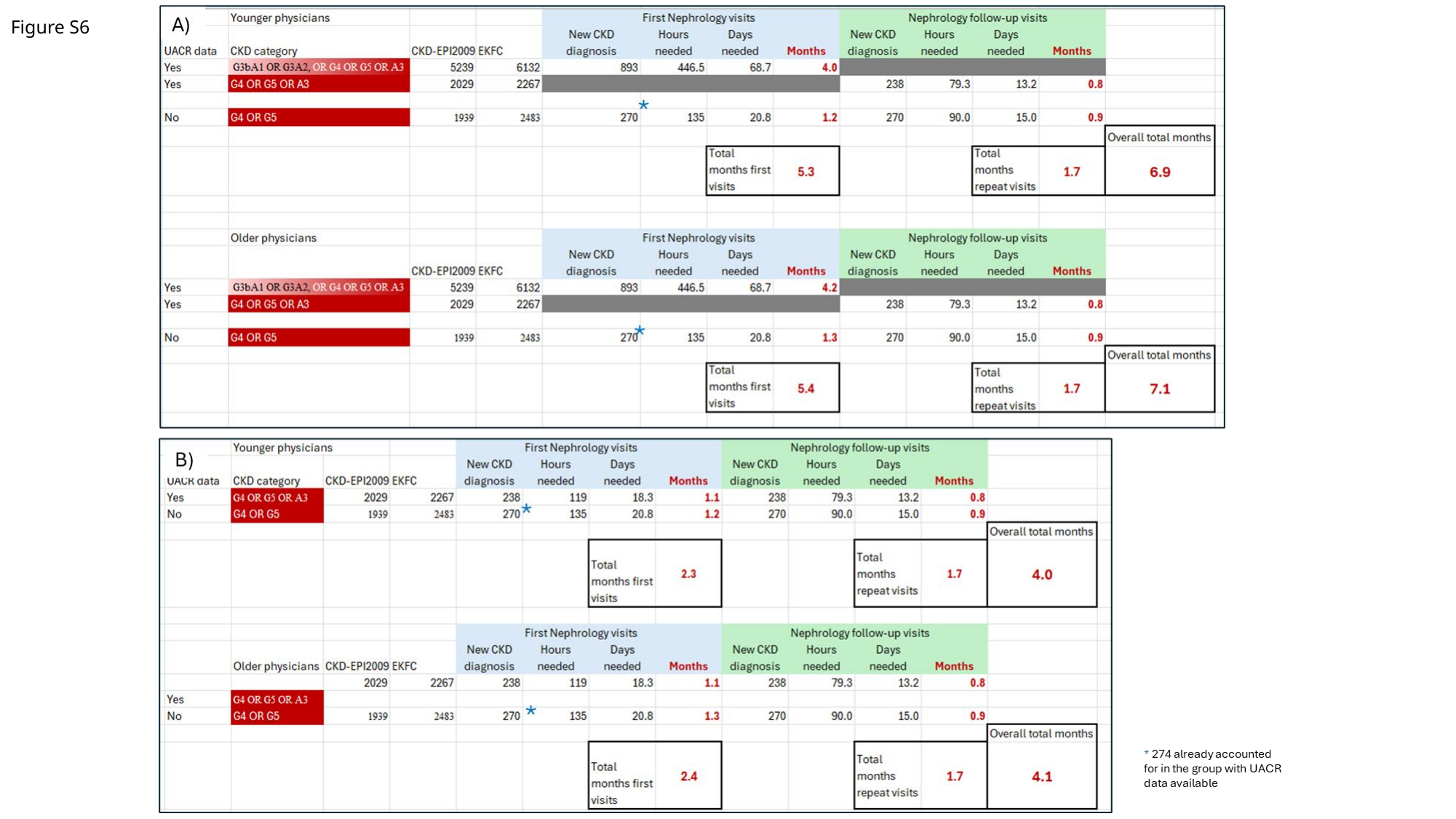

Figure S6

Supplement: sfaf278_Supplemental_Files [file sfaf278_supplemental_files.zip › Figure S6 R1 nm.pptx]
